# Asymptomatic leishmaniasis in kala-azar endemic areas of Malda district, West Bengal, India

**DOI:** 10.1371/journal.pntd.0005391

**Published:** 2017-02-10

**Authors:** Pabitra Saha, Swagata Ganguly, Moytrey Chatterjee, Soumendu Bikash Das, Pratip K. Kundu, Subhasish K. Guha, Tamal K. Ghosh, Dilip K. Bera, Nandita Basu, Ardhendu K. Maji

**Affiliations:** 1 Department of Microbiology, Calcutta School of Tropical Medicine, Kolkata, West Bengal, India; 2 Department of Zoology, A. P. C. Roy Govt. College, Himachal Bihar, Matigara, Siliguri, West Bengal, India; 3 Department of Microbiology, N. R. S. Medical College & Hospital, Kolkata, West Bengal, India; 4 Malda Medical College, Malda, West Bengal, India; 5 Department of Tropical Medicine, Calcutta School of Tropical Medicine, Kolkata, West Bengal, India; 6 Medinipur Medical College, West Medinipur, West Bengal, India; 7 Calcutta School of Tropical Medicine, Kolkata, West Bengal, India; Institut Pasteur de Tunis, TUNISIA

## Abstract

Asymptomatic leishmaniasis may drive the epidemic and an important challenge to reach the goal of joint Visceral Leishmaniasis (VL) elimination initiative taken by three Asian countries. The role of these asymptomatic carriers in disease transmission, prognosis at individual level and rate of transformation to symptomatic VL/Post Kala-azar Dermal Leishmaniasis (PKDL) needs to be evaluated. Asymptomatic cases were diagnosed by active mass survey in eight tribal villages by detecting antileishmanial antibody using rK39 based rapid diagnostic kits and followed up for three years to observe the pattern of sero-conversion and disease transformation. Out of 2890 total population, 2603 were screened. Antileishmanial antibody was detected in 185 individuals of them 96 had a history of VL/PKDL and 89 without such history. Seventy nine such individuals were classified as asymptomatic leishmaniasis and ten as active VL with a ratio of 7.9:1. Out of 79 asymptomatic cases 2 were lost to follow up as they moved to other places. Amongst asymptomatically infected persons, disease transformation in 8/77 (10.39%) and sero-conversion in 62/77 (80.52%) cases were noted. Seven (9.09%) remained sero-positive even after three years. Progression to clinical disease among asymptomatic individuals was taking place at any time up to three years after the baseline survey. If there are no VL /PKDL cases for two or more years, it does not mean that the area is free from leishmaniasis as symptomatic VL or PKDL may appear even after three years, if there are such asymptomatic cases. So, asymptomatic infected individuals need much attention for VL elimination programme that has been initiated by three adjoining endemic countries.

## Author summary

A total of 79 asymptomatic VL cases were detected from two kala-azar endemic blocks of Malda districts of West Bengal by active mass screening. Follow study of the asymptomatic cases revealed that 10.39% cases transformed into disease and 9.09% cases remained as sero-positive even after three years. So progression to clinical disease among asymptomatic individuals was taking place at any time up to three years after the baseline survey. Therefore, in a given period if there are no cases of VL/PKDL for two or more years, it does not mean that the area is free from leishmaniasis as symptomatic VL or PKDL may appear even after three years, if there are such asymptomatic cases. So, asymptomatic infected individuals need much attention for VL elimination programme that has been initiated by three adjoining endemic countries.

## Introduction

Visceral leishmaniasis (VL) is a vector-borne protozoal disease caused by the *Leishmania donovani* species complex. Estimated 200000–400000 new cases are recorded per year worldwide [1] of which 67% of the total cases are contributed by three Asian countries India, Nepal, and Bangladesh. In this part of the world *L*. *donovani* is the only causative agent of VL, *Phlebotomus argentipes* is the only known vector and the only reservoir is human [2, 3]. Availability of reliable, effective and safer drugs like oral miltefosine and liposomal amphotericine for treatment, indoor residual spray for vector control and antigen based rapid diagnostic test kits (rK39) usable at field level makes the disease as a candidate for elimination [4, 5].

On this background India, Nepal, and Bangladesh launched a joint VL elimination initiative in 2005 with a target of bringing the incidence down to less than 1 case per 10,000 population by 2015 [6]. This elimination initiative faced an important challenge due to asymptomatic infection. *L*. *donovani* infection results into a full-blown symptomatic disease or asymptomatic carrier without any clinical manifestations for a long period.

Asymptomatic leishmaniasis is not well defined; generally it is described by a positive serological test, polymerase chain reaction (PCR), or leishmanin skin test (LST) in individuals who are apparently in a healthy condition [7, 8].

In an endemic area, most of *L*. *donovani* infections remain asymptomatic [9]. Cross-sectional surveys based on serological studies showed that a high proportion of serologically positive persons are asymptomatic [10–17]. Mathematical modeling shows that asymptomatic carriers, constituting a reservoir of parasites, may drive the epidemic in future [18], although their infectiousness to sand flies is yet to be established.

Several prospective studies have documented the ratio of asymptomatic infection to incident clinical VL cases as 1:2.4 to 5.6:1 in African countries [19–21], 18:1 to 50:1 in South American and European countries [22, 23] and 3.8:1 to 8.9:1 in Asian countries [24–26]. The role of these asymptomatic carriers in disease transmission, the prognosis of these cases at the individual level and the rate of transformation to symptomatic VL/PKDL needs to be evaluated. The present study was designed to determine the actual burden of the asymptomatic cases and pattern of disease progression (development of signs and symptoms of VL or PKDL), and rate of sero-conversion (serologically positive to negative) among them in an endemic area of Malda district, West Bengal.

## Materials and methods

### Study area and population

**Fig 1:**
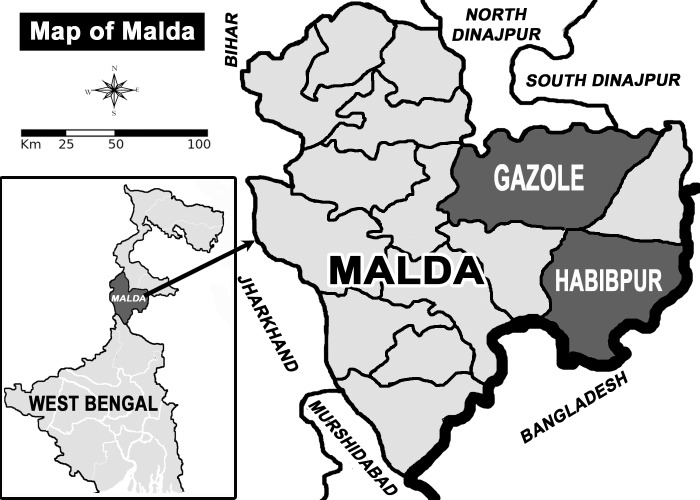
Map showing the study sites.

Depending upon the records of State/District health offices about VL/PKDL cases during past few years, eight tribal villages of three sub-centres of Malda district were selected. The district Malda is situated just north to the river Ganges; it has an international border with Bangladesh in the East and an interstate border with Bihar in the West which is endemic for leishmaniasis (Fig 1).

### Mass survey

Before initiation of the mass survey population of the villages were sensitized and explained about the objectives of the study and were requested to participate. Volunteers were selected from the population and trained about the study protocol and grouped into three member teams leaded by one person from project staff / an investigator from Calcutta School of Tropical Medicine. The teams made complete census of the study population by house-to-house visit and filled up a questionnaire in a printed book, about history of VL/PKDL and treatment received, age, sex, presence / history of fever, occupation, level of education. After obtaining verbal consent from the head of the family and also from each individual, rapid diagnostic test (RDT) based on rK39 was done for the detection of anti-leishmanial antibody.

All RDT positive persons were examined clinically for the detection of probable cases of VL/PKDL. Suspected such cases were subjected for parasitological investigation and referred to their local primary health centres (PHCs) for treatment. Apparently healthy person positive for antileishmanial antibody were classified as asymptomatic cases and were followed up to record the development of any sign of VL or PKDL and to study the pattern of sero-conversion during three study years.

### Ethical consideration

The ethical aspect of the study was described earlier [35]. In brief, the objectives of the study were explained to the village population by the study team headed by a medical doctor from Calcutta School of Tropical Medicine. The selected population was invited to participate into the study. They were also informed that their identity would not be disclosed in any way and they could withdraw from the study at any time without any explanation. Written informed consent was obtained from antileishmanial antibody positive cases or from their legal guardians of the child below the age of 14 years, having no history of kala azar detected by mass screening. There were three such cases between the age group of 14 to 17 years and informed written consent was obtained from them in presence of their legal guardians. Such cases were examined clinically for detection of sign and symptoms of active VL and asymptomatic VL cases were followed up for three years. The Ethics Committee of the Calcutta School of Tropical Medicine reviewed and approved the study protocol.

### Follow-up of asymptomatic cases

All the identified asymptomatic VL cases were followed up both clinically and serologically on 3^rd^, 6^th^, 12^th^, 18^th^, 24^th^, 30^th^ and 36^th^ month of the study to know the rate of progression to clinical disease and sero-conversion.

### Treatment of diagnosed VL cases

All diagnosed VL and PKDL cases were treated with either Sodium Stibo Gluconate (SSG) or miltefosine as per National Vector Born Disease Control Program (NVBDCP) of India. Drugs were supplied by Dy. Director of Health Services, Malaria, State Programme Officer, Government of West Bengal.

## Results

### Prevalence of asymptomatic leishmaniasis in study areas

The study was undertaken during September 2012 to August 2015 in eight tribal villages of Malda district, West Bengal. In all villages under study, the houses are made of mud or brick. The source of drinking water in those villages is tube well and household works are done by pond water. Most of the houses have cattle sheds. Pig rearing is the common practice in all villages. Most of the peoples are agricultural or migratory labour by profession. Total population of the study village was 2890 (1439 male and 1451 female) among them 2603 population was screened for antileishmanial antibody. A total of 185 individuals were positive for antileishmanial antibody, of them 96 had a history of kala-azar/PKDL and 89 without any history of kala-azar (KA). Individuals (n = 89) having no past KA history were examined clinically for fever/ history of fever, pallor, hepato-splenomegaly by clinicians of the team. Ten such individuals were diagnosed as active VL and were treated with sodium stibo gluconate (SSG) as mentioned earlier. Remaining 79 cases were classified as asymptomatic leishmaniasis with a prevalence of 3.16% (79/2497) among the healthy individuals, of them 43 was male and 36 female. The demographic parameters of asymptomatic cases are given in Table 1. The ratio of asymptomatic infection to incidence active VL cases was 7.9:1 at that time in the study population.

**Table 1:** Demographic parameters of asymptomatic rK39 positive cases.

Characteristics	n = 79
**Sex: no. (%)**	
Male	43 (54.43)
Female	36 (45.57)
**Age category: no. (%)**	
1–5 yrs	15 (18.99)
6–14 yrs	19 (24.05)
≥ 15 yrs	45 (56.96)
**Age: Yrs**	
Mean	22.04
Range	1–65
SD	± 17.31
95% CI	18.16–25. 92
Sero-conversion rate: no. (%)*	
Became sero-negative after 1 yrs	0
Became sero-negative after 2 yrs	14 (18.18)
Became sero-negative after 3 yrs	48 (62.34)
Remains sero-positive after 3 yrs	7 (9.09)
Disease developed	8 (10.39)
Development of disease: no. (%)*	
within 6 months	1 (1.3)
within 6 months—1 yrs	2 (2.6)
within 1–2 yrs	1 (1.3)
within 2–3 yrs	4 (5.2)

* Loss to Follow-up = 2.

### Follow up of asymptomatic cases and pattern of sero-conversion and disease progression

**Fig 2:**
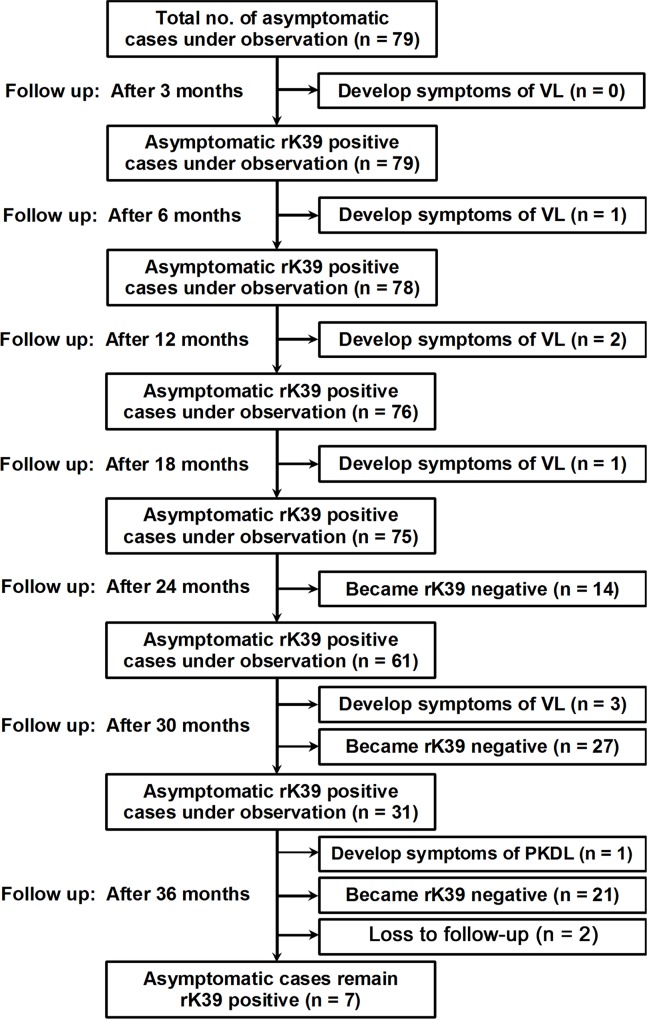
Flow chart showing the follow-up of asymptomatic VL cases and their sero-conversion pattern.

Healthy individuals having no signs and symptoms of VL or PKDL but positive for antileishmanial antibody were classified as asymptomatic infection. Among the asymptomatic cases who became serologically negative during follow up period were referred as sero-conversion and those developed the signs and symptoms of VL or PKDL as disease progression. All asymptomatic individuals (n = 79) were followed up both clinically and serologically at a regular interval of six months for three years to determine the rate of sero-positive to sero-negative and also to record the progression from asymptomatic infection to symptomatic VL or PKDL. During 36 months of follow up visit, 2 asymptomatic cases were lost to follow up as they moved to other places. Symptoms of VL developed in 7 (9.09%, 7/77) asymptomatically infected individuals, one after 6 months, two after 12 months, one after 18 months, three after 30 months. During 36 months follow up, sign of PKDL was observed in one asymptomatic case which was parasitologicaly confirmed by the demonstration of amastigotes in skin scrapping smear. The patient was examined thoroughly but did not show any symptoms of VL—like fever, history of fever during last one month and splenomegaly. A total of 62 (80.52%) cases became sero-negative, 14 after 24 months, 27 after 30 months and 21 after 36 months follow up period. Interestingly 7 (9.09%) individuals still remained positive serologically even after three years. So they need more follow up period. The patterns of sero-conversion and progression to clinical disease of the asymptomatic individuals are given in Fig 2.

### Treatment of VL cases

Out of 17 VL case, 10 were treated with SSG and remaining 7 with miltefosine (MIL). All the patients responded to their respective chemotherapy provided and no sign of PKDL was recorded among them during three years of the study.

## Discussion

The development of full blown VL/KA from the initial infection by the bite of an infected sand fly is a complex phenomenon and it takes few months to years. Several factors like host’s immunity [27] and nutritional status [28, 29] play an important role in it. In an endemic area a large number of individuals are asymptomatic with positive for antileishmanial antibodies. These individuals are apparently healthy and are not attending any health care system for diagnosis. The rate of such infection varies from region to region. In the present study we observed a prevalence rate of 3.16% (79/2497) asymptomatic leishmanial infection among the healthy population screened which is lower than the range of 5.62% - 13.8% detected by a no. of studies in Bihar using rK-39-ICT [30]. The ratio of asymptomatic infection with symptomatic cases was 7.9:1. Similar ratio of asymptomatic infection was also reported from India and Nepal [25], whereas a lower ratio was also reported from other parts of India and Bangladesh [24, 26]. A higher ratio was also reported from Brazil and Spain [22, 23] which might be due to different parasite species i. e. *L*. *infantum* with distinct life cycle to that of *L*. *donovani* in India. This ratio of asymptomatic infection to disease may differ from village to village within the same population has been reported by Khalil et al., 2002 [31] due to different risk factors like contact of VL, presence of other seropositive cases, family size, house type, cattle rearing and poverty [30].

India, Nepal, and Bangladesh took an initiative to eliminate VL by 2015 from this part of the World [6]. Asymptomatic infection is one of the great challenges faced by this initiative. Several questions remained unresolved regarding these asymptomatic infections—whether these persons are infectious to the sand flies? Who among the asymptomatically infected people will develop VL and when [14]. Which infection remains asymptomatic and how long [27]. Population-based long term prospective epidemiological studies are needed to answer these.

In the present study we observed that 14 (18.18%) asymptomatic individuals became sero-negative after two years and 48 (62.34%) after three years of initial diagnosis. Most importantly 7 (9.09%) still remained sero-positive even after three years, which requires more follow up, but how long, it is difficult to say. In a prospective study in India and Nepal Ostyn et al., 2011 [25] observed a higher rate of sero-eversion (86.7%) among asymptomatic infections within one year. Similar observation was also made in Brazil and Kenya [22, 20].

In the present study, symptomatic VL developed in 7 (9.09%) asymptomatic cases during three years of follow up. The proportion asymptomatic VL infection that progress to clinical disease within one year was 3.9% which is within the range of 1.5% - 23.1% as recorded in Bihar studies [30]. A study from Bihar, India by Topno et al., 2010 [26] showed that clinical VL developed in 7 (18.42%) of the 38 asymptomatic individuals in less than 6 months of initial diagnosis. The rate of progression to disease was 17.85/1000 PY, similar observation was also made from another parts of India and Nepal [25] and also from Brazil [22, 32]. Topno et al., 2010 [26] also reported that no new cases of VL were developed after six months follow up of asymptomatic cases. In contrast, in the present study we recorded longer latent period for *L*. *donovani* infection. In addition 7 (8.9%) cases remained asymptomatic after three years. Our study showed that progression to clinical disease among rK39-positive asymptomatic cases taking place at any time, up to three or more years after the baseline survey which is similar to that observed by Gidwani et al., 2009 [14] in Bihar. Most important aspect is the development of PKDL in one of the asymptomatic cases after three years of initial diagnosis. PKDL is considered as a reservoir for transmission of VL [33–35] as the parasites are easily available to the vector sandfly. We have reported a high prevalence of PKDL from the same study area previously [36]. So long term prospective studies are required to observe the fate of all asymptomatic cases.

Scientists used different tools for diagnosis of asymptomatic infection like DAT, rK39 and PCR. In the present study we used rK39 based rapid diagnostic kit for this purpose. It is easier to perform at the field level without any invasive method and it does not required any sophisticated laboratory facilities like to that of PCR based diagnosis.

In a given period if there are no cases of VL/PKDL for two or more years, it does not mean that the area is free from leishmaniasis as symptomatic VL or PKDL may appear even after three years, if there are such asymptomatic cases. So, asymptomatic infected individuals need much attention for VL elimination programme that has been initiated by three adjoining endemic countries. If we allow these individuals to stay in the community, there might be a chance of epidemic in future. Though the available antileishmanial drugs are costly and toxic, but a case control study by treating all asymptomatic cases with single dose liposomal amphotericin in a given geographical area may enlighten the role of such intervention for elimination to eradication of leishmaniasis.

## Supporting information

S1 Checklist Strobe. (DOCX)
